# The Great Arcanum:—The Turkish Bath

**Published:** 1861-07

**Authors:** 


### Art. III.—THE GREAT ARCANUM:—THE TURKISH BATH.

Can it be that the *Great Arcanum,* in search of which active spirits of the Middle Ages plunged themselves into hopeless bewilderment, is no trick of the imagination?—that the *Elixir of Life* and its correlate the *Philosopher's Stone* are no fictions?—that to the wisdom of the nineteenth century is reserved the achievement of unveiling that transcendental mystery which the alchemy of a remoter period and the magic of all ages have in vain attempted to penetrate?—that we may at length clap our hands, and, with jocund voice, cry out "*Eureka*"?

Strange though it may seem, yet it is not less strange than true, that these questions are pertinent to a subject which, at the present time, is justly attracting no small interest among the profession and the public at large. Zealous advocates of the so-called Turkish Bath, which has recently arisen into notoriety among us, men of mark and fame, are boldly attributing to that form of bathing virtues which hitherto (all charlatanism being set aside) have been considered alone possible in the supposititious Elixir of Life. Think not we exaggerate; for we are taught by these authorities no less than this—that by opening the flood-gates of the skin from time to time, and submitting ourselves to the manipulations of a shampooer, in the Oriental fashion, we may attain unblemished health under all circumstances, securing ourselves from the attacks or the consequences of sickness, and controlling or obviating the ill effects of our evil passions. So long, it is said, as we diligently and regularly have recourse to the Oriental bath, neither malaria, nor miasma, nor infection—neither the poison of marsh-fever, nor city-fever, nor of any form of fever whatsoever, can find a lodgment within us. We may hold at bay the direst contagious disorders, or root them out from among us. Nought that is deleterious can keep a place in the system when the pores of the surface are called into full action. All that is hurtful is swept away in the health-giving, health-preserving perspiration which streams forth. We may snap our fingers at blighting scrofula, wasting consumption, and the racking pains of rheumatism and gout. We may rest in assured safety against any curmurring of the stomach and its associated organs. No blotch, no stain, not even the tiniest freckle shall ever spot our dainty skin; no spasm torture our brawny frame; no mischief steal into the chambers of the heart, or damage the conduits of the blood. The mind and the brain—nay, the whole nervous system, central and peripheral, shall hold their own against the deteriorating influences which haunt our daily life. Man, indeed, may confront the world, armed in proof at all points against every danger which may assail his frame, whether wholly or in detail, from morbific agencies, if he will but habituate himself to the use of the Turkish Bath.

Nay, more, if by ill hap, in an unguarded moment, disease should steal in upon us (for no one is cautious at all times), no other medicament is needed than the Bath. Its commoner action is depuration by the skin, but it also acts, we are told, by purgation and diuresis, and is effective as a narcotic and tonic if need be. It is, in fact, a complete *materia medica* in itself. But while the physician might hesitate or doubt, and administer his remedies at haphazard, the Bath operates without let or error, according to the strict necessity of the case; bringing about, intelligently, as it were, the precise remedial action required.*

If we are gluttonous, the Bath will counteract the evil effects of our over-gorging; if dissolute, of our debauchery; if intemperate, of our drunkenness; if slothful, of our sloth. The indolent man who systematically submits himself to the all-healing process can at once start from his indolence, and undergo extreme toil; and the exhaustion of fatigue, the pangs of hunger, the carking cares of life—all yield to its potent influence.

* "It is thus a drug," writes Mr. Urquhart, "which administers itself according to need, and brings no after consequences."—*The Pillars of Hercules*, vol. ii. p. 74.

Premature wrinkles, greyness, the decrepitude of age, and baldness, alike vanish in the Bath; from its use also the entire surface of the body becomes indifferent to changes of temperature, and we may pass heedlessly and harmlessly from a torrid to an arctic zone, and set at defiance all those insidious currents of cool air which are pregnant with catarrh and a host of annoying disorders. The sensitiveness of the skin to mechanical injury moreover diminishes, while at the same time a more refined touch is developed.

To dwellers in towns the Bath is an efficient substitute for air and exercise, and a sure method of securing perfect health and prolonging life; to dwellers in the country it is the proper and needful means of thoroughly utilizing the healthful advantages they possess. The Bath, of a truth, not only makes what is healthy more healthy still, but sweeps away all those disadvantages which are commonly thought to be inseparable from insanitary districts, by enabling the inhabitants if they will, with little trouble and less cost, to obtain and maintain a degree of health equal to that found in the most healthy localities.*

Finally, the use of the Bath is as conducive to the perfection of our moral as of our physical powers.

"Do you think I fable with you?" exclaims Mammon, in the *Alchemist*, speaking of the Elixir of Life—

"I assure you
He that has once the power of the sun,
The perfect ruby, which he calls elixir,
Not only can do that,† but, by its virtue,
Can confer honour, love, respect, long life,
Give safety, valour, yea, and victory,
To whom he will. In eight and twenty days
I'll make an old man of fourscore a child, . . .
Restore his years, renew him, like an eagle,
To the fifth age.

. . . . . . . . . . .

'Tis the secret
Of nature naturiz'd 'gainst all infections,
Cures all diseases, coming of all causes;
A month's grief in a day; a year's in twelve:
And of what age soever, in a month.
Past all the doses of your drugging doctors.
I'll undertake, withal, to fright the plague
Out o' the kingdom in three months."‡

---

* "It must always be borne in mind that the thermæ has even a wider sphere; the Londoner, or the inhabitant of a large city, would live as healthily immured within his city walls as the rustic amid the fields and meadows of the country. His thermæ would be to him in the place of a country house, of a horse; it would give him air, exercise, freshness, health, and life."—Wilson's *Thermo-Therapeia*.

† *i.e.*, produce gold at will.

‡ *The Alchemist*, act ii. scene I.

Now, not only does an extraordinary parallelism exist, as we may thus see, between the marvellous properties of the Elixir of Life and those attributed to the Turkish Bath, but there is also a very curious analogy between the preparation of the one, and the process of the other. Subtile, when describing his method of projection, says:—

"I exalt our medicine
By hanging him in *balneo vaporoso*,
And giving him solution; then congeal him;
And then dissolve him, then again congeal him:
For look, how oft I iterate the work,
So many times I add unto his virtue."*

And again,—

"*Subtile.*—Your Magisterium, now?
What's that?
"*Face.*—Shifting, sir, your elements,
Dry into cold, cold into moist, moist into hot, hot into dry.
"*Subtile.*— . . . Your *Lapis Philosophicus?*
"*Face.*—'Tis a stone, and not
A stone; a spirit, a soul, and a body:
Which if you do dissolve, it is dissolv'd;
If you coagulate, it is coagulated;
If you make it fly, it flieth."†

It will be granted that there is a remarkable correspondence in conception between the alchemical processes here referred to, and the different stages of the Turkish Bath. It may, indeed, be reasonably surmised that, supposing the virtues of the latter ultimately justify the assertions of its chief advocates, it will prove to be the *Great Arcanum,* so long and vainly sought for. We can conceive that the Alchemist, led astray by barbaric greed, erred by looking upon Gold as the chief and primary aim of existence, and seeking the prolongation of life solely for the purpose of enjoying riches; instead of regarding wealth as subsidiary to health. Hence he sought to obtain Gold directly by transmutation of the baser metals, and not indirectly by first endeavouring to promote the fullest vigour of life, and so increasing the capacity of winning riches. Thus he failed to perceive the fact, to us indisputable, that the Philosopher's Stone, which, as he rightly defined it, was "a stone, yet not a stone; a spirit, a soul, a body," was no elemental body, but simply Man himself, who contains within himself all the essentials of the true Elixir of Life. To educe these, therefore, in all their purity, it follows that it was Man that should have been put into the alembic and distilled, or the crucible and sublimed, and not base copper, or brass, or tin, or

* *The Alchemist,* act ii. scene 3. † Ibid. scene 4.

any other telluric or vegetal substance. This, we may now perceive, can happily be effected in the Turkish Bath, which may rightly be regarded, according to the fancy, and without unduly stretching the imagination, as an alembic or crucible, in which man is subjected to an elaborate process of distillation or sublimation.

Let it not be supposed that we are desirous of throwing ridicule upon the Turkish Bath by indicating the parallelism which exists between its method of operation and reputed virtues, and the method of preparation and the properties of the hypothetical Elixir of Life. We would merely hint the probability that an enthusiasm which approximates its object to the most mystical and least tangible of the alchymical and magical doctrines of the Middle Ages, will be apt to lead to a fate similar to that which has befallen those doctrines. We should deeply regret to see so promising a sanitary movement as that which is now going on for the establishment of Turkish Baths in this country, checked either by the disappointment of exaggerated hopes, or by the too hastily expressed opinions of medical or scientific men being made use of as stalking-horses for charlatanism. It would be idle to assert that the value of the Turkish Bath has been tested in any mode which merits the term of scientific, yet it is by strict observation alone that that value can be determined. We know certain general facts which serve conclusively to show that the use of this bath may prove hygienically of great value, and it is highly probable that in our hands it may become an important therapeutical agent; but at present, both in its hygienic and therapeutic uses, we have not got beyond the bounds of those familiar principles which have hitherto governed the application of hot air, hot vapour, and friction to the body. In facilitating and securing the full effect of these means, either in the preservation of health or in medicine, the Turkish Bath offers mechanical advantages which have never before been possessed in this country, and from the use of which much good may justly be augured; but not so much surely, until the matter has been more thoroughly tested, as would warrant us in elevating the Bath to the dignity of a panacea for all the physical ills of life.

More or less enthusiasm is perhaps inseparable from the use of the Turkish Bath, when once we have become habituated to its luxurious delights; and perhaps it is well that it should be so, if we would hope to secure fully its introduction among us. But still it seems to us that the patent advantages of the Bath are sufficiently great to warrant its adoption in this country, without straining the fancy unduly for arguments. Our own acquaintance with the Bath was formed, where it is, for the present at least, best formed, that is to say, in Turkey. We have now lying before us the notes that we made after our first introduction to its mysteries, and they may fittingly form a portion of this gossip of ours.

The scene is Stambul: the time early morning. The gutter-like and still deserted streets glow, as it were, oven-like, with the stored-up warmth of many hot days and nights. A slight motion among the luxuriant foliage, which here and there straggles above the walls that hem us in on either side, hints of a breeze coquetting with the blue waters of the Bosphorus and the Sea of Marmora, and provokes longings for a plunge amid the rippling waves, within sight of the snow-clad summits of the Mysian Olympus. But with expectation all a-gog, and imagination inflamed by travellers' stories, we hasten on to the great baths of Sultan Mahmoud, and in a few moments stand within the doorway. The echo of our footsteps, as we crossed the threshold, pleasantly traversed the faint splash of a fountain which occupied the centre of the entrance-hall. Two or three attendants alone were lounging there, and by them we were ushered into a species of gallery, in which we were speedily divested of our garments. A long, soft, white towel was cast kilt-wise about our loins, and another thrown over the shoulders. Then descending to the floor of the bath, we thrust our feet into the loop of a pair of primitive-shaped wooden pattens, and, steadied by two slim, dark-skinned, bath satellites, we hobbled to a door, which, being cast open, admitted us into a large dimly-lighted chamber, filled with watery vapour, and so heated that we literally gasped for breath. In a few seconds the sense of suffocation that had at first seized upon us passed away, and we were enabled to look about us comparatively at our ease. The chamber was capped by a dome; and ceiling, walls, and floor were alike constructed of marble—at one time probably white as the driven snow, but now mottled with many dark stains, and discoloured throughout. To the right and left of the entrance were raised platforms, also of marble, and upon these several of the Faithful were curled up, pipe in mouth, and be-towelled like ourselves. Presently, the perspiration starting from every pore, we were conducted into another, larger, and much handsomer chamber, also constructed of marble, which, from neglect and heat, had long been divested of its pristine beauty. A subdued light was admitted through several small circular openings in a lofty dome, beneath which was a slightly-raised platform. The walls of this chamber were interrupted by deep recesses, within which marble basins, supplied with hot and cold water from brazen taps, projected from the walls. One or two small apartments, provided like the recesses with the appurtenances requisite for completing the process of the bath, and in which privacy could be secured, communicated also with the great chamber. This was filled with a thin film of vapour, and heated to so great an extent that we became faint, and, but for very shame, we should have fled into a cooler atmosphere. Urged forwards by our attendants, we were led to the central platform, and the towel having been removed from our shoulders, and spread over the marble surface, we were directed to lie down. This we did, but immediately started up, truly in hot haste, so acute was the sense of scorching on our first impact with the floor. With a lofty courage, however, we again essayed to stretch ourselves on the fiery pavement, and, sooth to say, we now contrived bravely to support the first sensation of extreme heat. In a few moments, the skin becoming less sensitive, we were enabled to lie quietly on the back. The attendants, then, seeing that we had become amenable to the routine of the bath, or surmising that we were well-nigh helpless from exhaustion, and must perforce yield thenceforth unresistingly to their behests, left us to ourselves for a short space. We were prompted, at this stage, to exclaim with Antiphanes*—

> "Plague take the bath! just see the plight
> In which the thing has left me;
> It seems t' have boil'd me up, and quite
> Of strength and nerve bereft me."

In our case, however, we substituted the word broil'd for boil'd.

We had been laid upon the platform only a few seconds, when the perspiration streamed out of every pore in a marvellous fashion, and from moment to moment we became better able to endure the fervid atmosphere. Anon we managed to look without too much effort upon our fellow-bathers, several of whom were stretched near us, and others were undergoing more advanced processes of the bath in different recesses. It was pleasant and most encouraging to find that they appeared to be really enjoying themselves. Some were chatting and laughing cheerfully with the attendants, the countenances of others bespoke a more sedate delight, and a few even were singing, and ever and anon accompanied their song by striking their resounding flanks with open palm. The many-echoed chamber was filled with a pleasant din. One slim, lithe fellow droned out a most pathetic love ditty, in which he professed that his glowing affection for the loved-one was slowly reducing him to ashes. He might with more justice, we thought, have applied the idea to the scorching floor upon which he was then laid.

But now an attendant had once more taken his place at our side, and kneeling, he began gently to knead our limbs and body.

* Athenæus :—The Deipnosophists.

He applied himself to each limb in succession, then to the trunk; and finally, having contrived to give more or less motion to almost every joint in the frame, he helped us to our feet, and exclaimed, interrogatively, "Good?" Good, we were compelled to confess; for the whole of this process of shampooing was by no means unpleasant, and it was entirely divested of that violence which we had expected would, according to many descriptions, have been attached to it. We were next led across the floor into a recess, and directed to seat ourselves near one of the marble basins. Then our attendant, seizing hold of a metal laver which stood upon the edge of the basin, deluged us from head to foot with hot water. Next arming his right hand with a fingerless glove of camel's hair, he proceeded to chafe the moist and softened skin, carrying the gloved hand in long sweeps from the shoulder to the fingers, from the neck to the loins, and from the hips to the toes. At first the friction was applied gently; but by degrees the pressure was increased, until we experienced a lively sense of being flayed alive—an impression by no means assuaged by the sight of long rolls of effete scarf-skin rapidly forming beneath the glove. When the sense of flaying was at the height, the attendant suddenly started upon his feet and dashed a laver-ful of hot water over us. For a second or two we thought that we had been scalded, so painful was the sensation of heat, and our first impulse was to pitch the attendant head over heels as he stood, his face distended with a broad grin, eyeing our grimaces. We contented ourselves, however, as was best, with preventing him repeating the scalding affusion. Producing a wooden-bowl containing soap and a long wisp of vegetable fibre, he now dexterously whipped up a thick lather and anointed us with it from head to foot. Then thoroughly washing the soap from the head and shoulders with successive lavers-ful of warm water, he, with a graceful salutation, placed the vessel in our hands, that we might ourselves cleanse the rest of the body. After we had dashed water over our limbs to our heart's content, a towel was again wrapped about our loins and another thrown over our shoulders, while a third was entwined around our head turban-wise, and we were conducted once more into the middle apartment. Here, as may be imagined, descending as we were now from the torrid to the temperate zone of the bath, the atmosphere at first felt somewhat chilly. But this uncomfortable sensation presently passed away, and our attendant having removed superfluous moisture from the skin, by gently pressing upon it the towels with which we were clad, and having replaced these, now damp, with fresh ones, led us into the outer hall. We cast ourselves on the cushioned divan which surrounded the hall, glad to seek repose after the exhausting process we had undergone, and having imbibed a pleasant draught of cool sherbet, and received from the hands of a juvenile pipe-bearer a stupendous chibouque, we awaited with commendable patience the beatitude promised to those who had truly and faithfully submitted to the ordeal of the Bath.

Nor did we wait in vain. A delicious languor gently stole over us and gradually merged into a pleasing state of reverie, in which we experienced the apathetic and automatic delight peculiar to a condition of the most luxurious laziness. From this state we were ultimately aroused, by the presentation of a cup of black coffee, to a sense of physical well-being which was most agreeable, but which did not expel certain qualms of the stomach, which now prompted us to dress with what speed we might and hasten to the breakfast-table.

Subsequent experience mollified the more obnoxious phases of the Bath, and brought into fuller relief those which were more agreeable; and, in the end, when habituated to it, the whole process of bathing after the Turkish fashion became a most luxurious indulgence, particularly when we were fatigued either by bodily or mental exertion. But while admitting fully its virtues in this respect, we were far from convinced that it would be well if the Turkish Bath could be largely substituted in this country for other and much simpler forms of bathing. As an addition to those forms we could conceive that this Bath would be most valuable, as a substitute for them probably most detrimental. Thus, in the majority of cases, for ordinary purposes of cleanliness and vigour, the Turkish Bath, in our experience, by no means surpasses, and we question if it equals, in its beneficial effects the ordinary sponge, or shower, or plunge bath, whether warm or cold, with friction. This may, and ought to be, and is most beneficial when it is used daily; but the Turkish Bath cannot probably be made use of to its full extent more than once a week without giving rise to evil consequences—relaxing the whole system, and lowering the tone both of the body and the mind. But still we have much to learn on this point, for paradoxical though it may seem, we can obtain little dependable information upon the hygienic effects of the Bath from observation of the Turks themselves. Certainly their cleanliness, their excellent physical condition, their exemption from disease, and their temperance, have been paraded in connexion with the use of the particular form of bath which we commonly name after them. Our own acquaintance with the Turks, however, did not lead us to any very lofty notion of their cleanly habits in the strict sense of the term. Granting that by the use of the Bath at stated intervals, the Turk secures an average degree of bodily cleanliness greater than among ourselves, we were led to doubt not only the efficacy of the prescribed *daily* ablutions as means of cleanliness, but also whether the Turk had any correct idea of what cleanliness meant beyond the forms commanded by his religious customs. And even the mode in which these were carried out, in so far as domestic utensils and domestic usages are concerned, were so little in accordance with our English notions of cleanliness, that we found it absolutely necessary, during several months' residence in Turkey, to insure by personal inspection such methods of "washing-up" and "cleaning-down" as particularly affected our personal comfort. For example, one peculiar matter of domestic contention was the use of special towels for special purposes. The rule of the Faithful appeared to be to use one towel for personal cleanliness, dusting furniture, wiping dishes, and cleaning stew-pans, and it was only by strict watchfulness we could induce them to apply special towels to special uses. One servant, who was with us many months, a splendid specimen of the pure Turk, from Angora, in Anatolia, and who, we flattered ourselves, we had thoroughly inducted into habits of cleanliness, contrived, even at the last moment of his service with us, to give a fresh illustration of Turkish notions of cleanliness. Taking up a tumbler, preparatory to drinking some water, we were disgusted to see at the bottom of the glass a huge bug. (Bugs and fleas, we may remark parenthetically, are the legitimate occupants of the walls of wooden houses in the East). We pointed out the intruder to our faithful Omar, and directed him to eject it, and clean the vessel, when, to our horror and grief, he quietly dislodged the insect with his finger, and then proceeded to wipe the glass with a dirty pocket-handkerchief which he produced from some mysterious fold of his dress.

The best idea of the true value of a Turk's notion of cleanliness is perhaps obtained by observing the Turkish soldier in the field. There, when in presence of the enemy, he is exempted from strict attention to his religious ablutions. Under such circumstances he is, as a rule, except when he has the good fortune to be well officered, as filthy a being as could well be conceived both in person and generally, even when a plentiful supply of water may be at hand. Nay, so little are the notions of cleanliness engrained in him, that he will commonly irretrievably pollute the fountains from which his supplies of water are obtained. The British soldier stands out princely in respect of cleanliness, at least, in the field, compared with the Turkish. The Turk, in fact, from being usually cleaned by others in the Bath, never acquires properly the habit of cleaning himself.

To estimate the part which the Bath plays in securing the physical vigour of the Turks is a difficult task, if not one which cannot be satisfactorily performed. For in considering the question, it is not to be forgotten that the Turk abstains usually from all spirituous fluids, and is temperate in his diet—two causes most powerfully conducive to health, and acting contemporaneously with the Bath. Mr. Urquhart, however, has told us that we are "not to run away with the idea that it is Islamism that prevents the use of spirituous liquors: it is the Bath." But as the practice of cleanliness among the Moslems is governed by their religious creed, and the use of spirituous liquors is as strictly forbidden by that creed as cleanliness is enjoined, we may be permitted to doubt Mr. Urquhart's assertion. Moreover the use of the Bath is no preventive to the increasing use of intoxicating liquors among Turks of the higher classes, which seems to be taking place in consequence of their more intimate associations of late with less temperate nations.

During the Crimean War the Turkish soldiers, who were commanded by English officers, and regularly fed, maintained a higher degree of health than the English soldiers, even when the health of the latter was at the best. But, before we get to the question of the probable influence of the Bath in bringing about this result, we should first have to determine the influence exercised by age and one or two of the commoner domestic habits of life. The Turkish soldiers, as a rule, were men of maturer age and powers than the English soldiers, and consequently better able to bear the vicissitudes of campaigning. The youthfulness of too many of the latter was one of the most important causes of sickness during the war. Again, the food of the Turkish soldier in the field, when regularly issued, differed not a whit either in character, in amount, or in manner of cooking, from that which he received in garrison or that which he had when at his own home. At the best, the rations of the English army could not be stated to approximate so nearly to the food which he was usually accustomed to, and very commonly they differed widely from his ordinary diet. Life in tent or in quarters presented, also, but a slight difference from the domestic habits of the Turks, as compared with the domestic habits of the English. Finally, the Turk was temperate, the English soldier most intemperate. But, notwithstanding the advantages in favour of the Turks, if Turkish and English soldiers of the same ages were compared, he would have been a bold man who would have asserted that the Turks were the more brawny men. If, however, the sponge-bath-loving English officers were compared with the hot-air-bath-loving Turkish officers, the latter were seen to be as inferior to the former in physical development and vigour and beauty as in mental; yet the serious odds of great temperance in food and drink were commonly against the Englishman.

But who can measure the vast difference between the psychical condition of the Englishman and the Turk? It has often occurred to us that the mental and physical inertia which underlies, and is, indeed, the essential element of that luxurious state of lazy reverie which is the most immediate consequence of a Turkish Bath, is the type of the normal condition of the Turk, mentally and bodily. Becker, writing of the bath of the ancient Romans, has well said, "Perspiration and appetite, which the earlier generations obtained by corporeal exertion and agricultural labour, were attained by a later race, that lived for the most part in idle inactivity, by means of sudation and hot baths."* This observation may not inaptly be applied to the Turks of the present day, whose present state is certainly a curious comment upon the opinions of those who would hold the Turkish Bath *per se* as being a means sufficient of itself for the physical regeneration of a nation.

The foregoing observations on the Turks are intended simply as hints that it is unsafe to rest any opinions of the hygienic value of the Turkish Bath upon deductions derived from its effects upon the Turks alone, and, we may safely add, their co-religionists.

Of the therapeutic value of the Bath we have still almost everything to learn; but when it is broadly asserted that it is a potent preventive of malaria, fevers, and pestiferous diseases, we would ask how comes it to pass that, in the region of the Turkish Bath, plague is endemic, and malaria as deadly as elsewhere? It may be that the commoner varieties of fever are perhaps not so frequent in the Turkish towns as in our own cities, but there are many links to find in the chain of reasoning before the exemption can be connected with the use of the Bath.

But it may be asked, after all these hints, and inuendos, and doubts, and difficulties, what, then, is the true value of the Turkish Bath? Has it, in truth, any special value at all? The real fact of the matter is, that these questions have to be determined in the Occident and not in the Orient, and an important step to their proper solution, the Bath (thanks, in the first place, to Mr. Urquhart) being now fairly among us, is to get rid of all that orientalism of speech which has contrived to approximate its virtues to those which have hitherto been conceived to be peculiar to the *Elixir of Life* alone.

As a means of thorough cleanliness, and for rousing a torpid

---

* *Gallus*, p. 327. "Thus [we quote from Becker] Columella judged of his time; and after mentioning Cincinnatus, Fabricius, and Curius Dentatus, complains:—*Omnes enim patresfamiliæ falce et aratro relictis intra murum correpsimus, et in circis potius ac theatris quam in segetibus et vinetis manus movemus. Mox deinde, ut apte veniamus ad ganeas, quotidianum crudidatem Laconicis sitim quærimus, noctesque libidinibus et ebrietatibus, dies ludo vel somno consumimus ac nosmetipsos ducimus fortunatos, quod nec orientem solem vidimus, nec occidentem.*"

system into action, it may well be conceived that this form of Bath would prove a great boon to many dwellers in towns who lead sedentary lives, to those who suffer from brain-fag, and to the workers in several dusty and dirty trades. Leaving the question of the comparative hygienic merits of the Turkish Bath with other forms of bathing undecided, we would note this great mechanical advantage belonging to the former—to wit, that its proper application may be more efficiently secured than that of any other form of bath, from the necessity of the process being carried out, when rightly performed, by an attendant, the bather remaining passive. The mechanical advantages of the Bath, both in construction and method of operation, also suggest that it may become of great use in the treatment of several diseases, which it is not needful to recapitulate here. But when it is asserted that the Bath will prove a *substitute*, under any circumstances, for air and exercise, and counteract the effects of intemperance, debauchery, and gluttony, we must have somewhat sounder arguments than mere physiological reasons, however plausible these may be. We cannot, we confess, put much faith in arguments which ultimately rest, as these do, upon a chemical and electrical theory of life. The reasoning brings too forcibly to mind the learned Mr. Shandy, Senior's, profound dictum, that "The whole secret of health depends upon the due contention for mastery betwixt the radical heat and the radical moisture." "You have proved that matter of fact, I suppose," quoth Yorick. "Sufficiently," replied Mr. Shandy. "Now could the man in the moon"——you know the rest, dear reader.

Let us, by all means, if we can get them, have Turkish Baths attached to all our public baths, as additions to, not substitutes for, them, and to every hospital, general or special; but until we have tested their merits somewhat more severely, let us not assign to them virtues which would warrant the conclusion that the Turkish Bath is the Great Arcanum unveiled. That it may prove so we sincerely hope, but we hope doubtingly.

